# Proteomics and transcriptomics analyses of ataxia telangiectasia cells treated with Dexamethasone

**DOI:** 10.1371/journal.pone.0195388

**Published:** 2018-04-02

**Authors:** Michele Menotta, Sara Orazi, Anna Maria Gioacchini, Chiara Spapperi, Anastasia Ricci, Luciana Chessa, Mauro Magnani

**Affiliations:** 1 Department of Biomolecular Sciences, University of Urbino "Carlo Bo", Urbino, Italy; 2 Department of Clinical and Molecular Medicine, Sapienza University, Rome, Italy; Cornell University, UNITED STATES

## Abstract

Ataxia telangiectasia (A-T) is an incurable and rare hereditary syndrome. In recent times, treatment with glucocorticoid analogues has been shown to improve the neurological symptoms that characterize this condition, but the molecular mechanism of action of these analogues remains unknown. Hence, the aim of this study was to gain insight into the molecular mechanism of action of glucocorticoid analogues in the treatment of A-T by investigating the role of Dexamethasone (Dexa) in A-T lymphoblastoid cell lines. We used 2DE and tandem MS to identify proteins that were influenced by the drug in A-T cells but not in healthy cells. Thirty-four proteins were defined out of a total of 746±63. Transcriptome analysis was performed by microarray and showed the differential expression of 599 A-T and 362 wild type (WT) genes and a healthy un-matching between protein abundance and the corresponding gene expression variation. The proteomic and transcriptomic profiles allowed the network pathway analysis to pinpoint the biological and molecular functions affected by Dexamethasone in Dexa-treated cells. The present integrated study provides evidence of the molecular mechanism of action of Dexamethasone in an A-T cellular model but also the broader effects of the drug in other tested cell lines.

## Introduction

Ataxia Telangiectasia (A-T) is a rare genetic syndrome caused by mutations in the ataxia telangiectasia mutated (ATM) [1] gene. The gene product codes for a protein kinase belonging to the PI3 Kinase-like Kinase (PIKK) [2]. Depending on the level of the mutation, the resultant loss of ATM protein expression or function can lead to pleiotropic clinical phenotypes [3] such as ataxia, oculocutaneous teleangiectasias, immunodeficiency, infections, radio sensitivity and proneness to cancer and neurodegenerative disorders. Typically, A-T patients are wheel-chair dependent by the age of ten, and their life expectancy is around twenty-five years. The ATM gene ensures DNA repair in the nucleus [4], while its role in the cytosol is still poorly understood [5–7].

No effective disease-modifying treatment is presently available, and supporting therapies are used to care for patients. However, in the last few years, observational studies [8,9] and clinical trials [10–12] have shown that treatment with glucocorticoids improves symptoms and neurologic functions in patients with A-T.

In spite of their efficiency, the mechanism of action of glucocorticoids in A-T subjects remains unclear. Hence, several studies have been carried out seeking to gain insight into the likely molecular action of glucocorticoids in A-T patients. The authors of the present study have previously described the influence of Dexamethasone on gene expression, splicing, NRF2-mediated antioxidant response by redox balance improvement and cellular nano-mechanics by cytoskeleton and nuclear dynamics [13–18]. D'Assante et al. have reported the influence of Betamethasone on molecules involved in autophagosome degradation [19].

The main aim of the present study was therefore to add to this body of knowledge regarding the mechanism of action of glucocorticoids in A-T, which may in turn lead to improvements in A-T patient therapies. Here in we examine the combination of two “omics” approaches (proteomic and transcriptomic) adopted to study lymphoblastoid cell lines (LCLs) treated with Dexamethasone. The modulated proteins and genes that were discovered were employed in a functional network analysis in order to evaluate the cellular molecular functions and biological processes influenced by Dexa action. The investigation was also extended to a wider sample size, allowing us to explore the variability of Dexa effects in different cell lines. Transcriptomic data were also compared with available *in vivo* data recently published [18].

## Material and methods

### Cell cultures

The lymphoblastoid cell lines (LCLs) used in this study were obtained from A-T patients (ATM^-/-^ AT129RM, AT50RM, ATK13RM, ATK36RM) and a healthy donor (ATM^+/+^ WT238). The cell lines WT238, AT50RM and AT129RM derived from a previous work [17], while the cell line ATK13RM and ATK36RM were isolated during a phase II clinical trial [11] with the approval of ethical committee and all patients provided informed consent (along with the consent of their parents or legal guardian, as required). The LCLs were maintained in RPMI1640 medium supplemented with 2 mmol/l L-glutamine, 50 mg/ml gentamycin and 10% fetal calf serum in 5% CO2 at 37°C. Cells were treated with 100nM Dexa for 48h prior to protein and RNA extractions. Dimethylsulfoxide (DMSO) was used as the drug vehicle and thus administered in untreated cells used as controls.

### Sample preparation and 2DE analysis

The 2DE analysis were performed on the AT129RM and WT238 samples. A total of approximately 1x10^7^ cells for each condition were washed in isotonic Tris/sucrose buffer and subsequently lysed in ice by sonication cycles in lysis buffer (50nM Tris-HCl, 150mM NaCl, CHAPS 0.5%, SDS 0.1%) containing protease and phosphatase inhibitors. After 20’ incubation, 15U of Benzonase was added and incubation was continued for an additional 30’. After a further sonication cycle in ice, the lysates were clarified by centrifugation. Proteins were precipitated by Acetone/TCA (4/1 volumes), washed in Acetone and dried. The pellets were re-suspended in Protein Extraction Reagent Type 4 (SIGMA) and after the protein concentration assay, 1mg was further diluted in the same buffer containing pH 3–10 ampholytes, 5mM TBP and loaded onto IPG ReadyStrips pH 3–10 NL (Bio-Rad), rehydrated at 50V for 12h at 20°C. Isoelectric focusing was performed on the protean IEF Cell (Bio-Rad) as follows: 15’ at 250V, rapid voltage ramping to 10,000V and a final step at 10,000V up to 80,000V hours. After equilibration and alkylation, the strips were laid on an 8–15% T gradient SDS-PAGE gel. The runs were performed at 20°C at constant current (each gel at 8mA for 60’, followed by 16mA until the run was completed). The staining had been previously performed by Brilliant Blue G–Colloidal (SIGMA) and then switched to a modified silver staining as described by Shevshenko and Mortz [20,21]. The analysis was performed in triplicate and there was a fair reproducibility between the replicates.

### Image analysis and LC-MS/MS

The image of each gel was acquired by Fluor-S MAX Multi-Imager scanner (Bio-Rad). Spots were detected, matched and quantified by Melanie software. Spot selection was performed on specifically altered protein abundance in AT129RM then on WT after the drug treatment. The spots were qualified as differentially abundant with a fold change >1.5 and p≤0.05, and were subsequently selected for MS analysis. Briefly, the spots were excised and processed as reported by Shevchenko and colleagues [22]. The resulting peptides were processed by the LC-ESI-MS/MS system (Q-TOF Micro^TM^ Micromass, Manchester, UK) equipped with a Z-spray nanoflow electrospray ion source and a CapLC apparatus. A Symmetry C18 nano column (Waters, Milford, Mass, USA) was employed as an analytical column.

The instrument was set in a positive ion mode using N_2_ as the carrying gas. The capillary was set to 2,800 V, the sample cone to 30 V and desolvation temperature to 80°C. The survey scan mode was set as follows: MS range from 200 to 1,500m/z, MS to MS/MS by ion intensity, MS/MS range from 200 to 2,000m/z. Collision energy was set according to the ions’ charge state using Argon as the collision gas. For protein identification, MS/MS spectra were used as query in MASCOT (Matrix Sciences, London UK). Protein identity was assessed (in addition to MASCOT score) with at least three-peptide coverage and consistency with pI/Mw inferred by 2D-PAGE.

### Western blot analysis

The antibodies (anti-HSPA8, anti-AIF, anti-14.3.3 ζ/δ, anti-Calreticulin, anti-HMGB1, anti-HPRT1 and Anti-PP2A A subunit) used in this study were from Cell Signaling Technology and were used as recommended by the supplier. Cell lysates were prepared from all available cell lines. After PBS washing, the pellets were re-suspended in Protein Extraction Reagent Type 4, and following sonication in ice, the lysates were clarified by centrifugation. The protein contents were measured using the Bradford assay and 20 μg of each sample was used for SDS PAGE separation and subsequent transferred on Hybond-C membranes (GE Healthcare Life Sciences). Primary antibodies were detected by secondary HRP-conjugated antibodies using the ECL detection system (Advansta). Whole lane normalization was used for quantitative investigation, as previously described by Colella et al. [23] and Gürtler et al. [24]. Experiments were performed in quintuplicates and the statistical analysis was performed using the paired t-test.

### Affymetrix microarray analysis

Total RNA extracts were obtained from all used cell lines using an RNeasy Plus Mini Kit (QIAGEN). RNA labelling and hybridization were carried out according to the Affymetrix two-cycle target labelling protocol. For each experiment the cRNA was hybridized to Affymetrix HTA 2.0 Gene Chip Array.

The data analysis, after pre-processing at probe level (CEL files), were performed by RMA background adjustment, quantile method for normalization and median polish for summarization. The FKBP5, TMEM2 and NFIL3 gene expression were evaluated by qPCR (ThermoFisher TaqMan® Gene Expression Assays) through a 7500 Real-Time PCR System (Applied Biosystems).

The relationship between protein quantities and corresponding gene expression (AT129RM and WT238) was individually evaluated in the samples. For the functional annotation of DEGs, genes were selected by the Affymetrix TAC console, using an FDR p-value ≤0.05 and the subsequent network analysis was performed by the Reactome (FI) Functional Interaction Network plugin for Cytoscape [25]. Alternative splicing analysis was also performed for the same two samples. The expressed genes with at least one differentially expressed PSR or Junction (FDR p-value ≤0.05 and splicing index >|2|) were considered as alternatively spliced.

The gene expression analysis was extended to all A-T samples by MeV [26,27]. Statistically and differentially expressed genes were selected by the paired permutation t-test (FDR≤0.01) and were further used to compute the hierarchical tree (HLC).

## Results

### Impact of Dexa on proteomic profile

In the past few years, we have carried out gene expression analysis of A-T cells after Dexamethasone treatment [13]. In the present study, we performed a proteomic comparative analysis of A-T and WT LCLs treated with Dexamethasone combined to a deeper gene expression and splicing examination performed by microarray.

**Fig 1:**
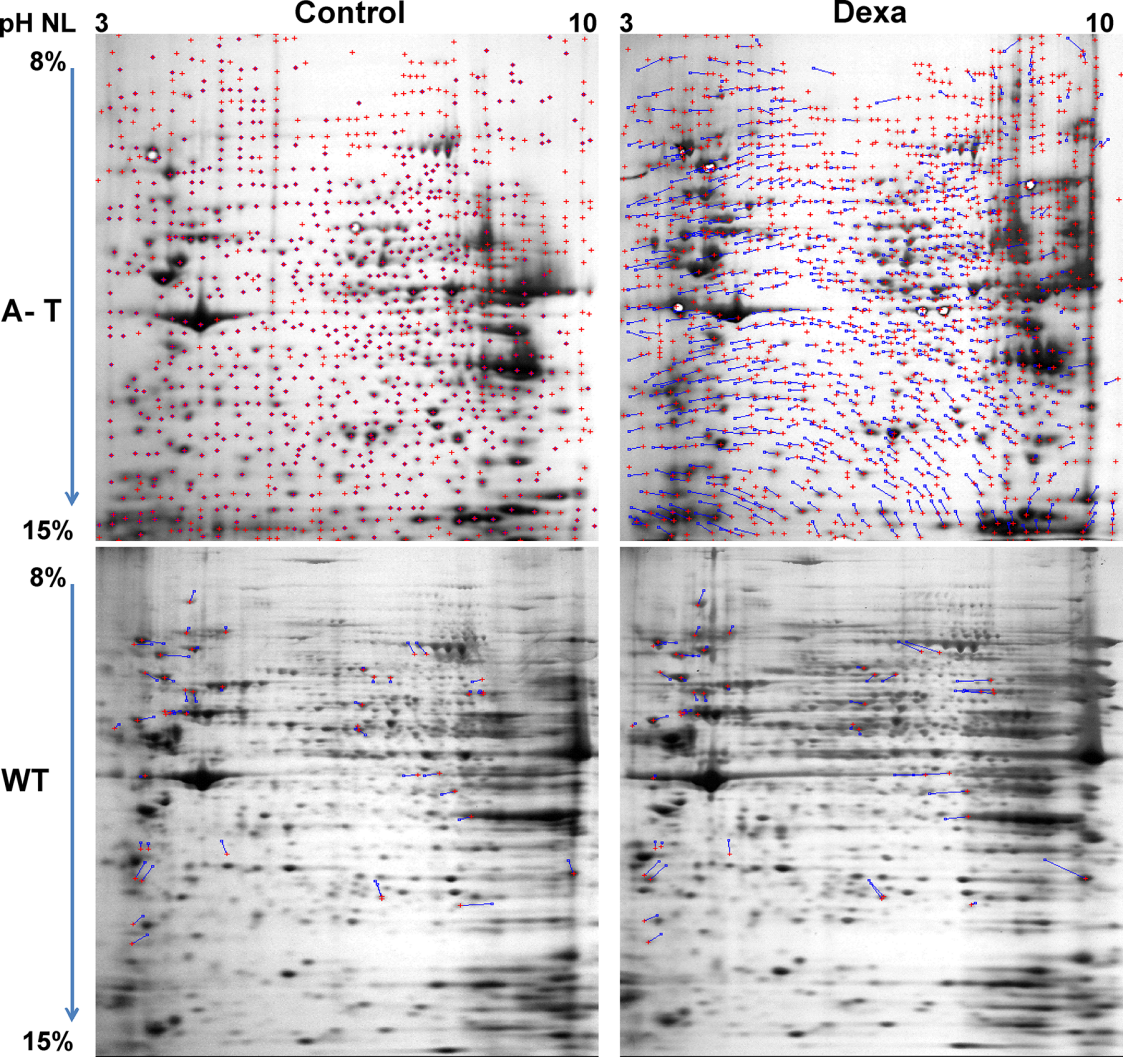
2DE representative images. A-T and WT samples treated or not treated with Dexa. Each gel image was elaborated with Melanie software. Three technical replicates were used.

Representative 2DE images are reported in Fig 1 (ATRM129, WT238 treated or not). Since the investigation mainly focused on those proteins that were specifically altered by Dexa in A-T samples, by comparing signal abundance in all samples, it was possible to isolate 52 spots out of 746±63. After processing, it was possible to define (due to handling and/or HPLC-MS failure) 34 differentially expressed proteins, reported in Table 1. As illustrated, the isolated spots were chosen since they were selectively and differentially altered in Dexa treated A-T samples than in treated or not WT. Even if a basal difference between the A-T sample and the WT one is noticeable (AT129RM/WT238 “Protein U or D” column) the effect of Dexa, not only eventually restored the lacking between samples, but also improved the extent of response was statistically different when compared to treated WT (Table 1, AT129RM +Dexa/WT238+Dexa “Protein U or D” column). The gene expression of each protein-matching gene is also reported alongside the p values from the variance analysis and FDR scores.

**Table 1:** Proteins regulated by Dexa inferred by the 2DE experiments. All comparisons scores. The bold text column shows the protein ratio (“**U**” upregulated, “**D**” downregulated and “**=“** as unvaried) of the indicated comparison, while the plain text reports the gene expression ratio (U upregulated, D downregulated and “**=“** as unvaried), the variance analysis p value and the FDR p value.

	AT129RM / WT238				AT129RM + Dexa / AT129RM				WT 238 + Dexa / WT238				AT129RM +Dexa / WT238+Dexa			
Gene Symbol	Protein U or D	Gene Expr. U or D	p value	FDR p value	Protein U or D	Gene Expr. U or D	p value	FDR p value	Protein U or D	Gene Expr. U or D	p value	FDR p value	Protein U or D	Gene Expr. U or D	p value	FDR p value
**ACTB**	**D**	=	0.00	0.02	**U**	=	0.47	0.73	**=**	=	0.03	0.28	**U**	=	0.04	0.18
**AIF**	**=**	U	0.01	0.10	**U**	=	0.43	0.7	**=**	=	0.30	0.70	**U**	U	0.05	0.19
**ATP5B**	**=**	U	0.04	0.16	**U**	D	0.15	0.44	**=**	D	0.29	0.68	**U**	U	0.17	0.35
**CALR**	**U**	U	0.00	0.06	**U**	=	0.05	0.27	**D**	D	0.31	0.70	**U**	U	0.05	0.19
**CCT3**	**U**	U	0.00	0.02	**D**	=	0.97	0.99	**=**	D	0.13	0.50	**D**	U	0.01	0.11
**EEF2**	**=**	=	0.27	0.46	**U**	=	0.58	0.81	**=**	=	0.10	0.47	**U**	U	0.02	0.13
**ENOPH1**	**=**	=	0.72	0.83	**U**	=	0.77	0.9	**=**	D	0.61	0.87	**U**	U	0.71	0.82
**EZR**	**D**	=	0.25	0.44	**U**	U	0.02	0.21	**=**	=	0.43	0.79	**U**	U	0	0.06
**FKBP3**	**U**	=	0.60	0.75	**U**	U	0.05	0.29	**U**	=	0.95	0.98	**U**	U	0.43	0.59
**FUBP1**	**=**	U	0.00	0.05	**U**	=	0.76	0.9	**U**	=	0.08	0.42	**U**	U	0.01	0.1
**GAPDH**	**=**	=	0.00	0.06	**D**	=	0.01	0.16	**D**	=	0.02	0.08	**D**	=	0.01	0.09
**HCLS1**	**=**	=	0.30	0.49	**U**	=	0.01	0.12	**D**	D	0.15	0.54	**U**	U	0.12	0.29
**HMGB1**	**U**	U	0.02	0.11	**U**	U	0.11	0.39	**U**	=	0.04	0.33	**U**	U	0.07	0.23
**HPRT1**	**U**	=	0.50	0.67	**U**	U	0.1	0.37	**D**	=	0.73	0.92	**U**	U	0.64	0.76
**HSP90AB1**	**=**	=	0.02	0.04	**U**	=	0	0.03	**=**	=	0.01	0.19	**U**	U	0	0.04
**HSP90AB2P**	**=**	=	0.06	0.19	**U**	D	0.12	0.39	**=**	U	0.00	0.02	**U**	D	0	0.07
**HSP90B1**	**D**	U	0.00	0.00	**U**	=	0.42	0.7	**=**	=	0.00	0.12	**U**	U	0	0.01
**HSPA8**	**D**	U	0.01	0.08	**U**	=	0	0.01	**D**	=	0.50	0.82	**U**	U	0.02	0.12
**HSPD1**	**=**	U	0.00	0.00	**U**	=	0.07	0.31	**=**	=	0.02	0.25	**U**	U	0	0
**HYOU1**	**=**	U	0.00	0.02	**U**	D	0.13	0.41	**U**	D	0.07	0.39	**U**	U	0	0.04
**LCP1**	**=**	=	0.00	0.04	**U**	=	0.002	0.07	**U**	=	0.00	0.02	**U**	U	0.29	0.47
**MSN**	**=**	=	0.00	0.07	**U**	D	0.12	0.4	**U**	=	0.40	0.77	**U**	=	0.28	0.46
**MZB1**	**=**	U	0.27	0.46	**U**	=	0.96	0.98	**=**	=	0.79	0.81	**U**	U	0.67	0.79
**P4HB**	**=**	=	0.49	0.66	**U**	=	0.28	0.58	**U**	=	0.56	0.85	**U**	U	0.57	0.71
**PCBP1**	**=**	=	0.25	0.44	**D**	=	0.75	0.9	**U**	D	0.02	0.24	**D**	U	0.03	0.16
**PPP2R1A**	**=**	=	0.15	0.34	**U**	=	0.56	0.79	**=**	=	0.16	0.55	**U**	=	0.61	0.75
**PSMB9**	**=**	D	0.27	0.46	**U**	=	0.99	0.99	**=**	D	0.43	0.78	**U**	=	0.75	0.84
**RPS3A**	**=**	=	0.67	0.80	**U**	=	0.55	0.79	**=**	=	0.42	0.78	**U**	U	0.73	0.83
**SRSF1**	**U**	U	0.01	0.10	**D**	=	0.63	0.83	**=**	D	0.39	0.76	**D**	U	0.03	0.11
**SYNCRIP**	**D**	U	0.00	0.01	**U**	=	0.86	0.94	**=**	=	0.00	0.11	**U**	U	0	0.02
**TPM3**	**=**	U	0.41	0.60	**U**	=	0.71	0.88	**=**	=	0.82	0.95	**U**	U	0.34	0.52
**TUBA1B**	**=**	U	0.11	0.28	**U**	=	0.51	0.76	**=**	=	0.70	0.91	**U**	U	0.1	0.27
**XRCC6**	**D**	U	0.00	0.05	**U**	=	0.66	0.85	**=**	D	0.08	0.43	**U**	U	0	0.08
**YWHAZ**	**=**	=	0.91	0.95	**U**	=	0.06	0.3	**U**	=	0.78	0.93	**U**	U	0.23	0.41

**Fig 2:**
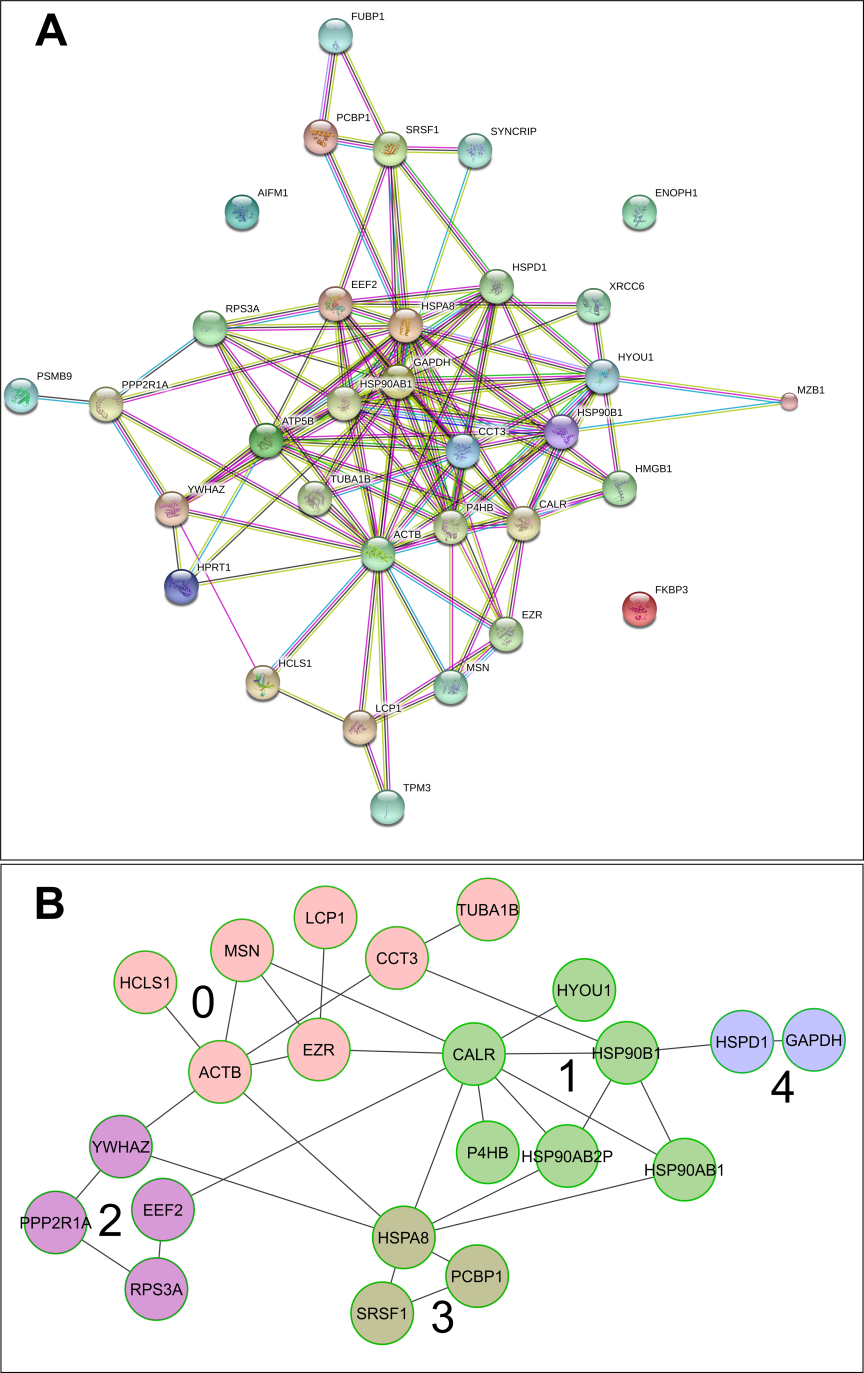
**(A-B) STRING (A) and Reactome FI (B) networks.** The proteomic data were used to compute both networks. The functional interaction of the discovered proteins in the AT129RM sample was extremely elevated as reported in S1 and S2 Supplementary Files. The nodes colours (in B) represent the Reactome FI clustered genes and the numbers state the enrichment pathways of nodes in clusters as reported in S2 Supplementary File.

The entire protein interaction network (PPI enrichment p-value = 0) based on the proteomic dataset is shown in Fig 2A. The corresponding biological processes, molecular functions, KEGG pathways, PFAM domains and INTERPRO features are reported in S1 Supplementary File, while the corresponding Reactome FI network pathways are reported in Fig 2B and described in S2 Supplementary File.

**Fig 3:**
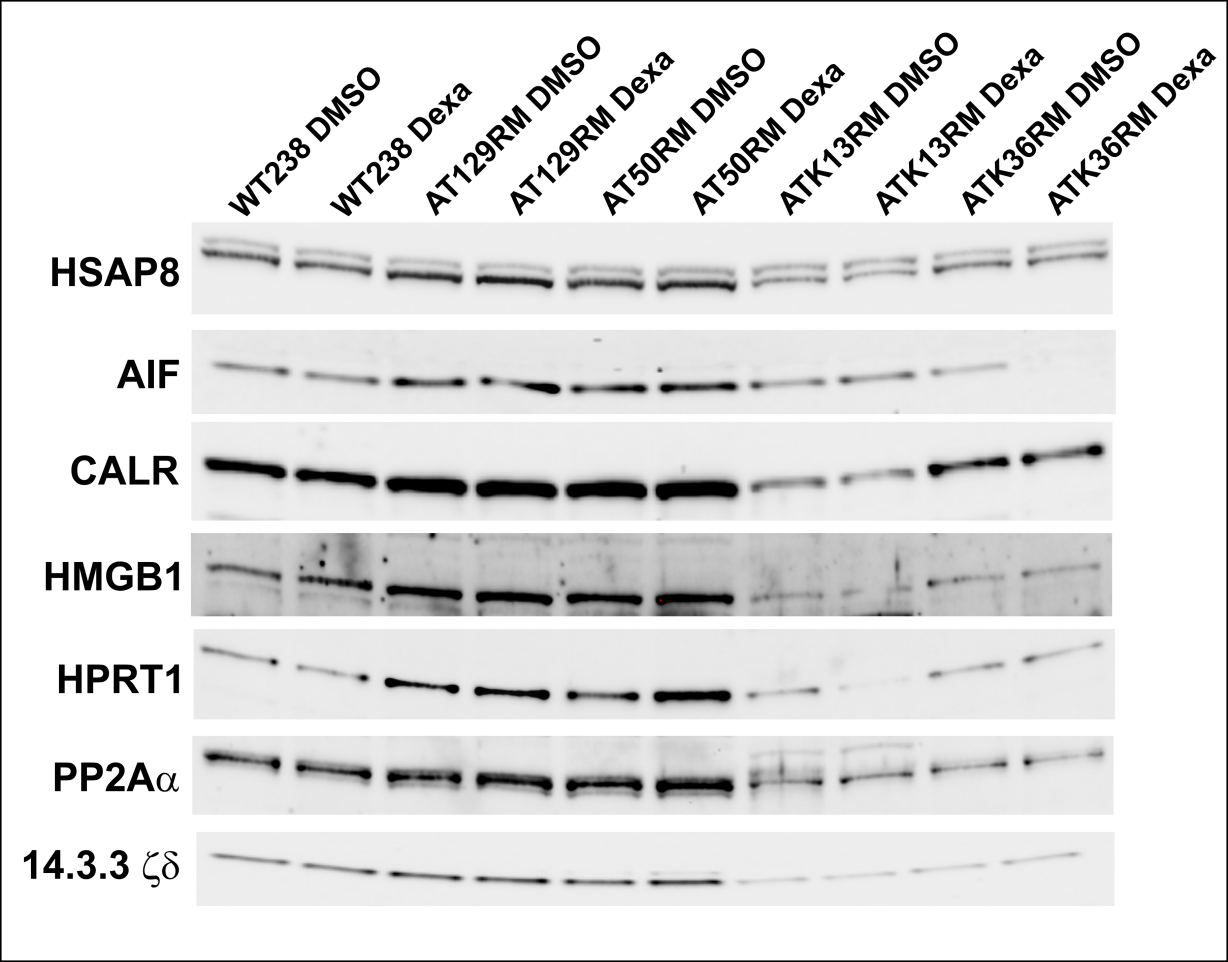
Western blot. Representative images of western blots performed in all tested LCLs, subsequently quantified as reported in Fig 4.

**Fig 4:**
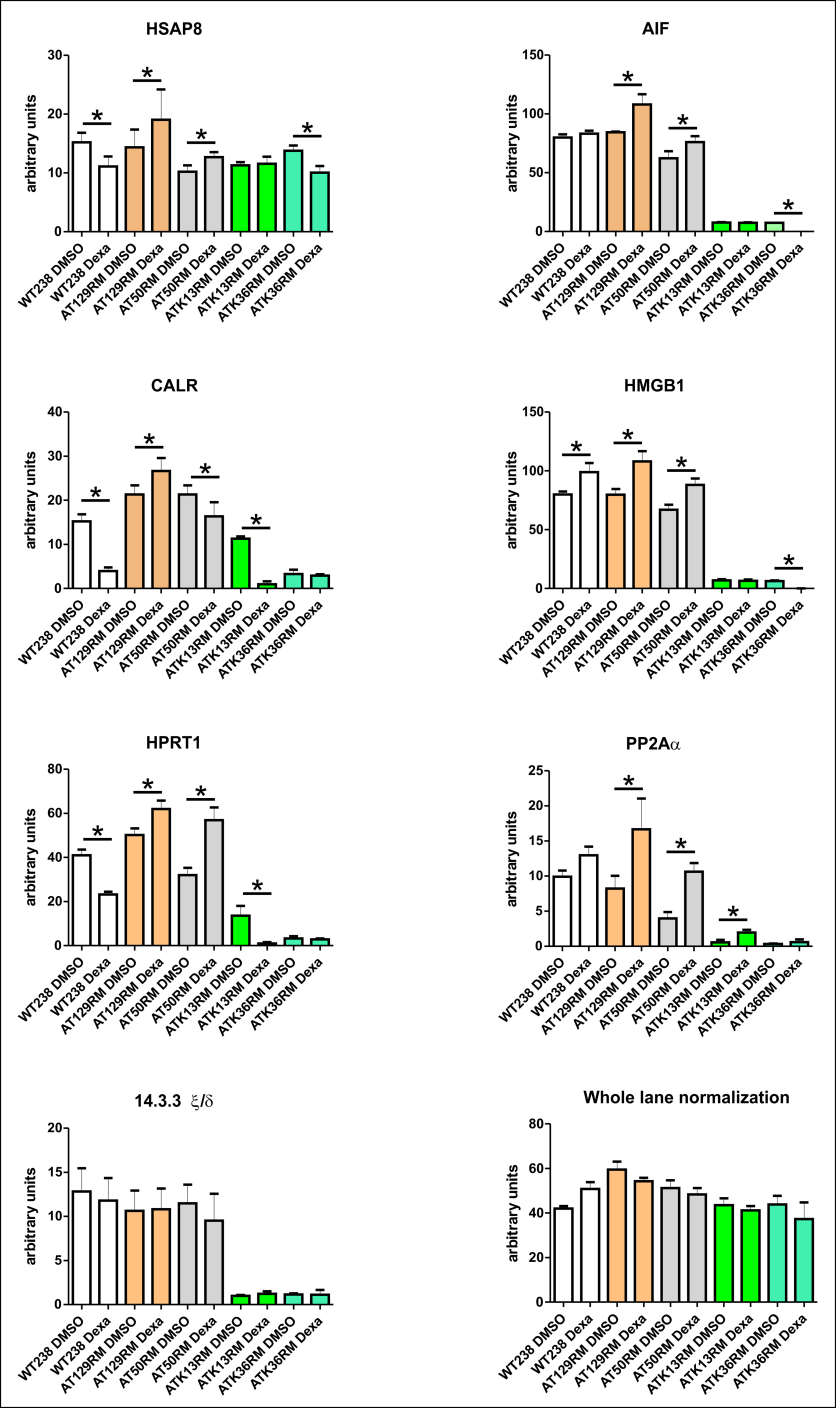
Western blot analysis of all tested LCLs. The protein abundance of selected targets in sample AT129RM is in agreement with the 2DE outcome (paired t-test p<0.05) except for 14.3.3 ζ/δ (see text). Only the AT50RM sample behaved in a similar manner to the AT129RM sample, despite the ATK13RM and ATK36RM cell lines. The W-N graphic reports the whole lane normalization data of the WB experiments.

The accuracy of the proteomic results was assessed by testing some randomly chosen targets (HSPA8, AIF, CALR, HMGB1, HPRT1, PP2A A subunit and 14.3.3 ζ/δ) using the western blot technique as reported in Fig 3 and Fig 4. All the tested western blots agree with the 2DE results (WT238 and AT129RM). Only the expression of the 14.3.3 ζ/δ protein differs from the 2DE data, as it appeared unaffected by Dexa. It should be noted that the protein assignment for the 2DE 14.3.3 spot was based on the maximum number of identified peptides in the Mascot outcome (14.3.3 ζ/δ definitely), but at least five isoforms were truly present in the isolated spot (supplementary S1 Fig). Accordingly, an erroneous antibody could be used, leading to the un-matching results of 2DE and the western blots.

### Impact of Dexa on gene expression profile

**Fig 5:**
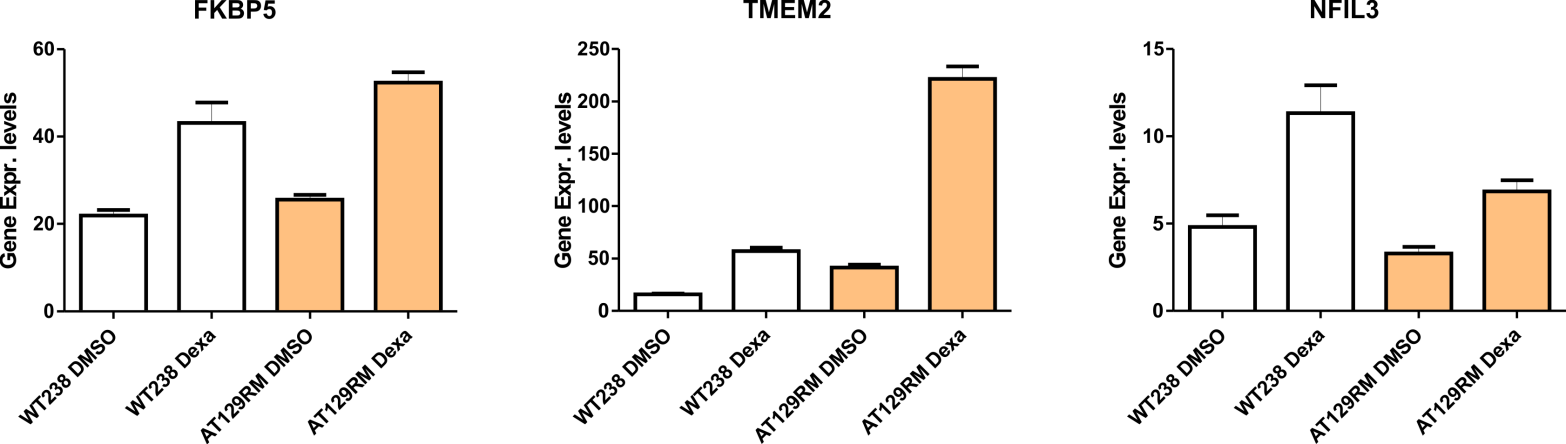
FKBP5, TMEM2 and NFIL3 gene expression by qPCR. The well-known genes altered by Dexa administration have been tested by qPCR in order to validate the microarray procedure.

The Dexa altered gene expression profiles of samples AT129RM and WT238 (599 and 362 gene symbols respectively) are reported in S3 Supplementary File, while the corresponding Reactome FI networks are reported in supplementary S2 Fig as well as the network pathways, biological pathways and molecular functions (S4 Supplementary File). The consistency of the microarray experiments was assayed by evaluating the gene expression of FKBP5, TMEM2 and NFIL3 targets, since they are well-known genes altered by Dexa administration both *in vitro* and *in vivo* [18,26–29]. In brief, the above-mentioned genes were actually found to be upregulated in all the microarray sets (treated samples over controls; FKBP5 average overexpression FC 2.2 p = 0.00 FDR = 0.05; TMEM2 average overexpression FC 5.5 p = 0.00 FDR = 0.07; NFIL3 average overexpression FC 2.11 p = 0.00 FDR = 0.8). The overexpression was confirmed also by qPCR performed on the same targets (Fig 5).

### Impact of Dexa on splicing

**Fig 6:**
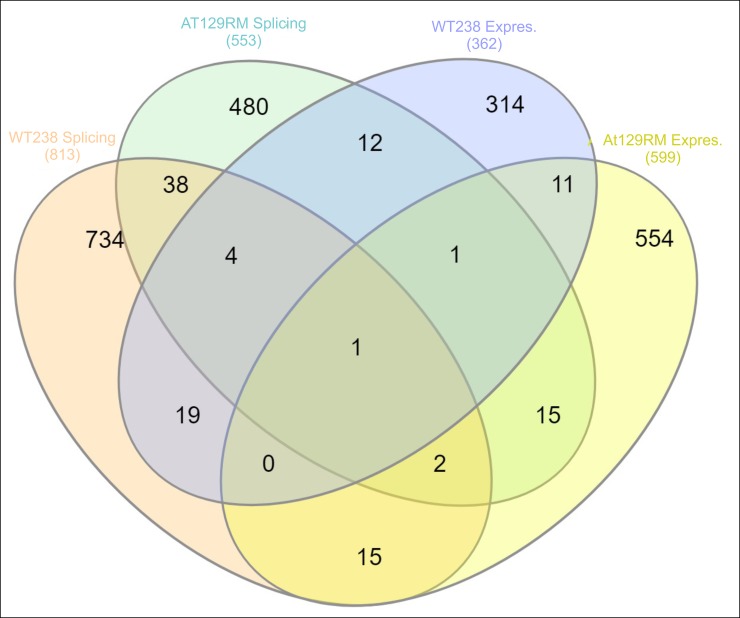
Veen diagram. The splicing and expression outputs were compared and plotted to show differences about spliced and altered expression genes between WT and AT. Only small amounts of gene symbols were shared in all tested comparisons.

The splicing analysis was performed by the Gene Chip Array dataset and 614 transcripts in AT129RM and 891 transcripts in WT238 (553 and 813 matching gene symbols respectively) were found to be alternatively spliced as reported in S5 Supplementary File. The affected biological pathways are reported in the same file, and the corresponding Reactome FI networks are illustrated in supplementary S3 Fig. Through the comparison of the splicing analysis gene sets and gene expression variations of both samples, it was possible to draw the Veen diagram reported in Fig 6. Only 19 gene symbols were found to be both modulated and spliced in AT129RM sample and 24 gene symbols in WT238 (only AP3S1 was in common, supplementary S1 Table).

### Survey on other A-T samples

**Fig 7:**
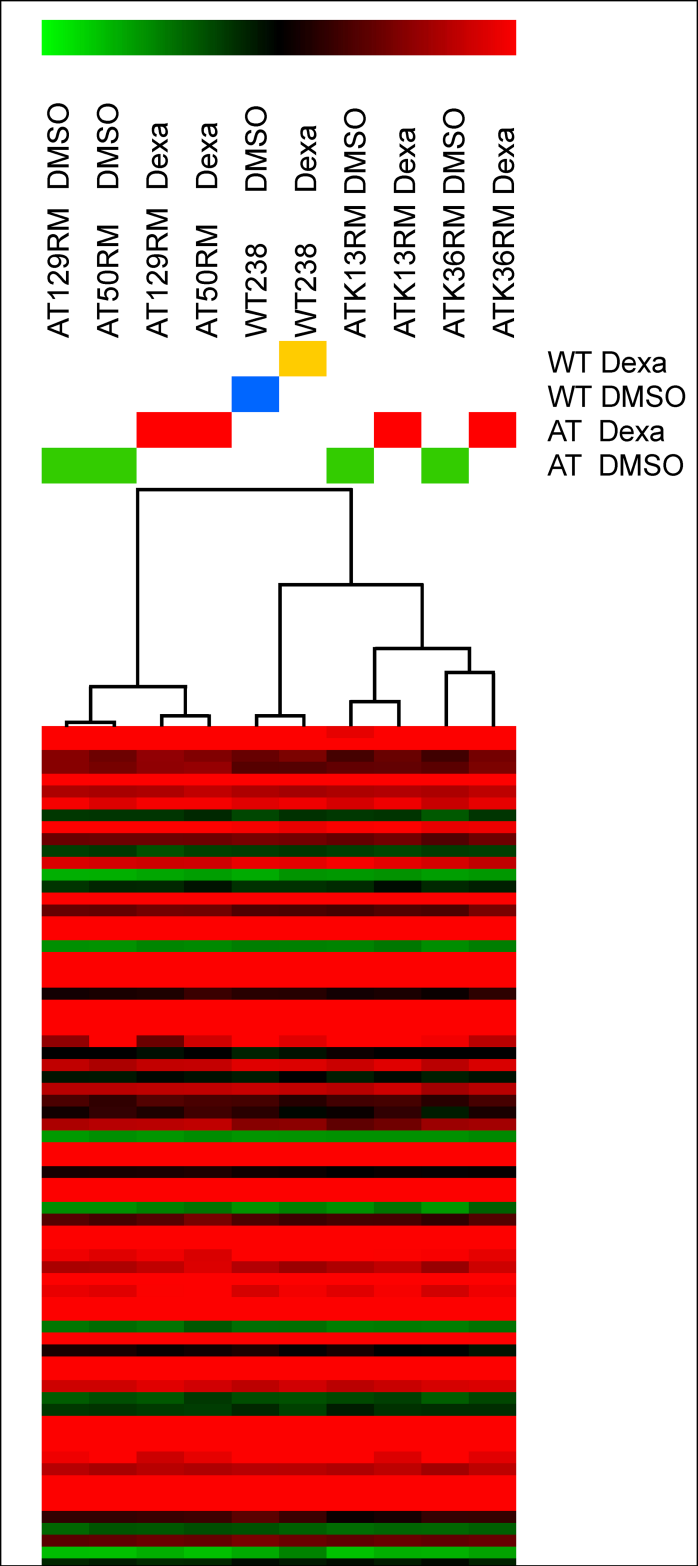
HLC outcome obtained by microarray expression profile of A-T samples. A total of 675 differentially expressed transcripts allowed us to classify AT129RM and AT50RM as similar to each other, while the other A-T samples behaved differently. The same behaviour pattern was inferred by western blot analysis.

All the tested antibodies (HSPA8, AIF, CALR, HMGB1, HPRT1, PP2A A subunit and 14.3.3 ζ/δ) were used to infer proteomic data of the other tested A-T cell lines as illustrated in the same Fig 3. Among the tested cell lines, only the AT50RM sample behaved like the AT129RM one. The other tested samples showed very low amounts of the investigated protein (except for HSAP8) with varied outcomes. The evaluation of gene expression on other A-T samples showed 675 statistically modulated transcripts (658 gene symbols) that were employed in the HLC clustering process reported in Fig 7.

The HLC dendrogram showed a similar expression pattern for the samples AT129RM and AT50RM and, separated by the WT238 group, the cluster of samples K13RM and K36RM shared a similar gene expression profile. The genomic data revealed similarity between the AT129RM-AT50RM and K13RM-K36RM samples. The gene expression features found in A-T samples statistically characterised the shared genes modulated by Dexa beyond the individual sample variability. The affected biological pathways are reported in S6 Supplementary File and the drawn Reactome FI network in supplementary S4 Fig.

## Discussion

Results illustrated in the present study represent the first integrated analysis by proteomic and transcriptomic, to investigate *in vitro* the Dexamethasone molecular mechanism of action in an A-T established model, as this drug is employed as effective therapy in A-T patients [11,12,16,18]. The chosen spots selection approach allowed highlighting the protein amounts variation in A-T cells in response to Dexa treatment, rather than the generalised cellular glucocorticoid response that, in our opinion, represent an attractive basis to comprehend the particular outcome of glucocorticoid usage in ataxia telangiectasia treatment. Some of the selected proteins in AT129RM showed also a basal dissimilar expression pattern compared to untreated WT (depending on A-T status?) but all of them showed a differential treatment response behaviour than WT. In fact, we were not able to observe any simple protein recovery in treated A-T.

Upon examining these results, we immediately noted the overall lack of correlation between gene expression and protein amount in the treated AT129RM sample. Surprising but acceptable if we consider that in the last few years several studies demonstrated the overall mRNA / protein correlation of about 0.4 [28–30].

In contrast, the same genes in the treated AT129RM/WT comparison showed a good gene expression-protein amount match. It is possible to suppose (within the sensitivity of microarray approach) that in the A-T sample, Dexa increased the level of the identified protein but not the corresponding gene expression while, at least after 48h of Dexa exposure, in the WT sample the drug affected the expression of the examined genes. Concerning the A-T sample, only the FKBP3 and EZR proteins showed a matching gene expression variation (according to the p value).

The FKBP3 gene expression might be modulated by Dexa in the same way that the FKBP5 gene is modulated. The codified protein has a role in DNA packaging, interaction with HDACs [31] splicing of mRNA, ribosomal assembly, along with other aspects of neuronal signalling [32,33]. The EZR gene has recently been reported to be induced by Dexa in podocytes [34] and to codify for an actin-binding protein involved in cytoskeleton reassembly. This last finding, together with the other identified cytoskeletal proteins discovered through proteomic analysis (ACTB, MSN, TPM3, CCT3, LCP1 and TUBA1B), support the findings of a previous work by the authors of the present investigation, in which the mechanical proprieties of the same cell line were found to be influenced by Dexa [15].

The overexpression of Calreticulin, a protein that binds Calcium, was particularly elevated in the AT129RM sample. It has a chaperonin-like activity and is able to bind transcription factors. Its regulation may be related to the effects of the glucocorticoids. In fact, it is able to interact with the DNA-binding domain of NR3C1, a receptor for glucocorticoids [35,36], and mediates its nuclear export [37]. Furthermore, CALR is involved in calcium storage in the endoplasmic reticulum, regulating diverse vital cell functions.

HSPA8 was found to be downregulated in WT238 and upregulated in AT129RM. This protein is also a molecular chaperone that is not just involved in protein homeostasis. Indeed, when interacting with other partners, HSPA8 is able to acquire other cellular functions [38], including a role in autophagy regulation [39], one of the biological paths affected in A-T cells.

In the proteomic derived Reactome FI network (Fig 2B), the following pathways were influenced: PI3K-AKT path, ATR signalling, spliceosome regulation and, as previously described, the regulation of the actin cytoskeleton. The stimulation of the AKT pathway by Dexa is in agreement with our previous observation [17] of AKT-ERK signalling activation. The role of AKT in DNA double strand break repair has been thoroughly described in the radioresistance mechanism of tumor cells [40–44], and we cannot exclude a similar behaviour in A-T cells as a balancing function for DNA repair. Furthermore, several studies associate the lack of ATM mediated AKT signaling with neuronal degeneration [45–47], and the possibility of a rescue mechanism promoted by Dexa is a very interesting prospect.

The HSP90 protein family (included in the reported proteomic data) is found in the PI3K-AKT node. The reported cytosolic HSP90AB1, HSP90AB2P and the ER HSP90B1 [48] may be related to the overall glucocorticoid effects on GR signalling, but interestingly, this family is also involved in DNA repair [49] and was reportedly impaired in A-T cellular models used in a previous proteomic study [50]. In the present investigation, Dexa was shown to improve the amount of these proteins.

Another protein directly involved in DNA repair is the identified protein XRCC6 (Ku70). This protein is known to participate in early time during the DSB damage response and can modulate the ATM-dependent ATR activation during this response [51–53]. It is noteworthy that in the analysed LCLs, the expression of miniATM was standing [17], and it could participate alongside the above-mentioned protein in the DNA repair process. Indeed, the presence of the term “ATR signalling pathway” (in the proteome derived Reactome FI network, S2 Supplementary File) and the highlighted biological pathways regarding DNA homeostasis (S4 Supplementary File, Reactome FI networks by microarray: Mitotic G1-G1/S phases, M/G1 Transition, DNA replication, Synthesis of DNA), suggest that the DNA repair process might be active in the case of DSBs.

Interesting the term “splicing” in the above-mentioned networks is often present and actually, by microarray, we were able to show that splicing occurred after Dexa exposure. It is also noteworthy that the ATM gene product was found to be alternatively spliced, which is consistent with findings of a previous report [17] by the authors of the present study. Since only 19 genes with altered expression were also spliced, all the other unaltered expressed but spliced genes probably contribute to confuse the whole outcome of Dexa effect in A-T cells, as illustrated by the influenced biological pathways of spliced gene list. Also in WT238 sample the splicing response was observed (24 gene products resulted both differentially expressed and spliced), but only 38 genes resulted commonly spliced as in A-T sample, suggesting that also the splicing response is differentially influenced by glucocorticoids in ataxia telangiectasia.

Cheema et al. [54] have reported proteomic profile changes in response to ionizing radiation (IR) in A-T cells and ATM complemented A-T cell lines. Interestingly, some of the identified proteins were also noted in the present investigation. In fact, Dexa proved capable of inducing the proteins ACTB, EEF2, EZR, FUBP1, GAPDH, MSN and SRSF1 in A-T cells in the same manner that they were induced in the IR exposure response. On the contrary, HSP90B1, HSPA8 and LCP1 were upregulated by Dexa, while they were downregulated in the case of IR stimulation. It could supposed that Dexa can partially simulate the radiation exposure.

The accuracy of the proteomic results was assessed by testing some randomly chosen by western blotting with a good data agreement, and by the same assay, it was possible to extend the proteomic data to the other tested samples. The behaviour of the other cell lines was inhomogeneous, and especially the cell lines K36RM and K13RM showed a different proteomic pattern compared to AT129RM and AT50RM, at least regarding the tested targets. In the last two samples, the amount of investigated proteins is lower than in all other cell lines and often they behaved completely different. This may be due to genetic variability of samples hence leading to a different response to glucocorticoids. The same matching correspondence was highlighted by HLC examination, thus suggesting that the proteomic pattern modulated by Dexa is actually influenced by the genotype of the tested cells, thus further puzzling the comprehension of a common molecular mechanism of action of Dexamethasone. Variation of Dexa efficacy was also noted in treated A-T patients [11] and the data illustrated may support the suggestion that response to Dexa is A-T subject genotype dependent. Unfortunately, we are not able to compare the tested cell lines to patient treatment outcome (concerning the cell lines K36RM and K13RM, derived from a clinical trial); the AT129RM and AT50RM samples are cell lines from ‘90 and no glucocorticoid therapy was ongoing. The authors of the present paper have recently reported the transcriptomic results of a clinical trial in which long term Dexa administration was performed in A-T patients using red blood cells [18]. Hence, the comparison of gene expression variation between *in vivo* data and the *in vitro* model was actually possible. The gene list of common modulated gene symbols is reported in supplementary S2 Table alongside the shared molecular pathways in the Reactome FI networks. There was only a slight overlapping of the results. This is probably due to the different biological samples used in the two studies, but undoubtedly the main differences lie in the administration modality, and hence the drug concentration, and effective exposure time. On the other hand, some biological effects were found both in *in vivo* and *in vitro*, such as the presence of the ATMdexa1 transcript [16]. Alongside the illustrated results, remains the puzzling effects of Dexa administration on the tested cell lines, assuming that the genetic variability exerts a significant influence on drug administration outcome.

Based on all of these findings we cannot rule out the possibility that the obtained proteomic data may also be extended to some *in vivo* biological responses. It would be very interesting to test some of the above-mentioned targets in the blood of A-T patients who will be enrolled in an upcoming phase III clinical trial (ATTeST, https://clinicaltrials.gov/show/NCT02770807).

## Supporting information

S1 Fig Mascot outcome of Spot #21. The protein assignment performed according to the highest number of covering peptides, in this case the 14-3-3 zeta/delta. However, the same spot also returned as 14-3-3 gamma, beta/alpha, theta and eta. The western blot unlatching results may be due to the different isoform of the tested antibody.
(TIF)

S2 Fig **(A-B)** Reactome FI networks derived by Dexa modulated genes in WT238 sample (A) and in A-T129RM (B). The gene expression analysis using the Affymetrix platform allowed us to isolate statistically and differentially expressed transcripts. The full list of differentially expressed transcripts is reported in S3 Supplementary File. The nodes colours represent the Reactome FI clustered genes while the numbers state the enrichment pathways of nodes in clusters as reported in S4 Supplementary File alongside biological pathways and molecular functions.
(TIF)

S3 Fig Reactome FI network of spliced transcripts. The STRING analysis of 2DE characterised spots from the A-T AT129RM sample lied to perform a splicing analysis of the same sample using the Affimetrix platform. Actually 614 transcripts proved to be alternatively spliced and were used to draw the functional network reported in **B**. Concurrently also the WT spliced transcripts were inferred and used to compute the functional network reported in **A.** The nodes colours represent the Reactome FI clustered genes while the numbers state the enrichment pathways of nodes in clusters as reported in S5 Supplementary File alongside biological pathways and molecular functions.
(TIF)

S4 Fig Reactome FI network by transcriptomic analysis of all A-T samples. The network details are reported in S6 Supplementary File. The nodes colours represent the Reactome FI clustered genes while the numbers state the enrichment pathways of nodes in clusters. The profile of all A-T allowed the HLC outcome and sample clusterization illustrated in Fig 7. The whole A-T transcriptome statistically would decrease the Dexa modulated genes variance due to the genetic variability of the samples.
(TIF)

S1 Supplementary File Excel file containing the STRING outcome of the protein highlighted by 2DE analysis in the AT129RM sample. (XLSX)

S2 Supplementary File Excel file reporting the Reactome FI outcome of the protein highlighted from 2DE of the AT129RM sample. (XLSX)

S3 Supplementary File Excel file containing the lists of differentially expressed transcripts in the WT238 and AT129RM samples. (XLSX)

S4 Supplementary File Excel file containing the outputs of Reactome FI analysis of microarray experiments. Gene symbols, nodes, paths in networks, biological pathways and molecular functions for samples WT238 and AT129RM are reported.
(XLSX)

S5 Supplementary File Excel file with the gene symbols list obtained by Affymetrix splicing analysis of the AT129RM and WT238 samples. Reactome FI nodes, paths in networks, biological pathways and molecular functions are also reported.
(XLSX)

S6 Supplementary File Excel file with microarray investigation of all A-T samples. Reactome FI nodes, paths in networks, biological pathways and molecular functions are described.
(XLSX)

S1 Table Comparison of the gene list from the splicing analysis with the gene expression variation in sample AT129RM (19 genes were found to be both modulated and spliced), and in WT238 (24 genes were both modulated and spliced). (DOCX)

S2 Table Gene list of common modulated gene symbols and shared molecular pathways in the Reactome FI networks from the comparison of gene expression variation between *in vivo* and *in vitro* data. (DOCX)
