# Supplementary figures and images for: Proteomics and transcriptomics analyses of ataxia telangiectasia cells treated with Dexamethasone
Source: PLoS One. 2018 Apr 2;13(4):e0195388. doi: 10.1371/journal.pone.0195388 (PMC5880408; doi:10.1371/journal.pone.0195388)

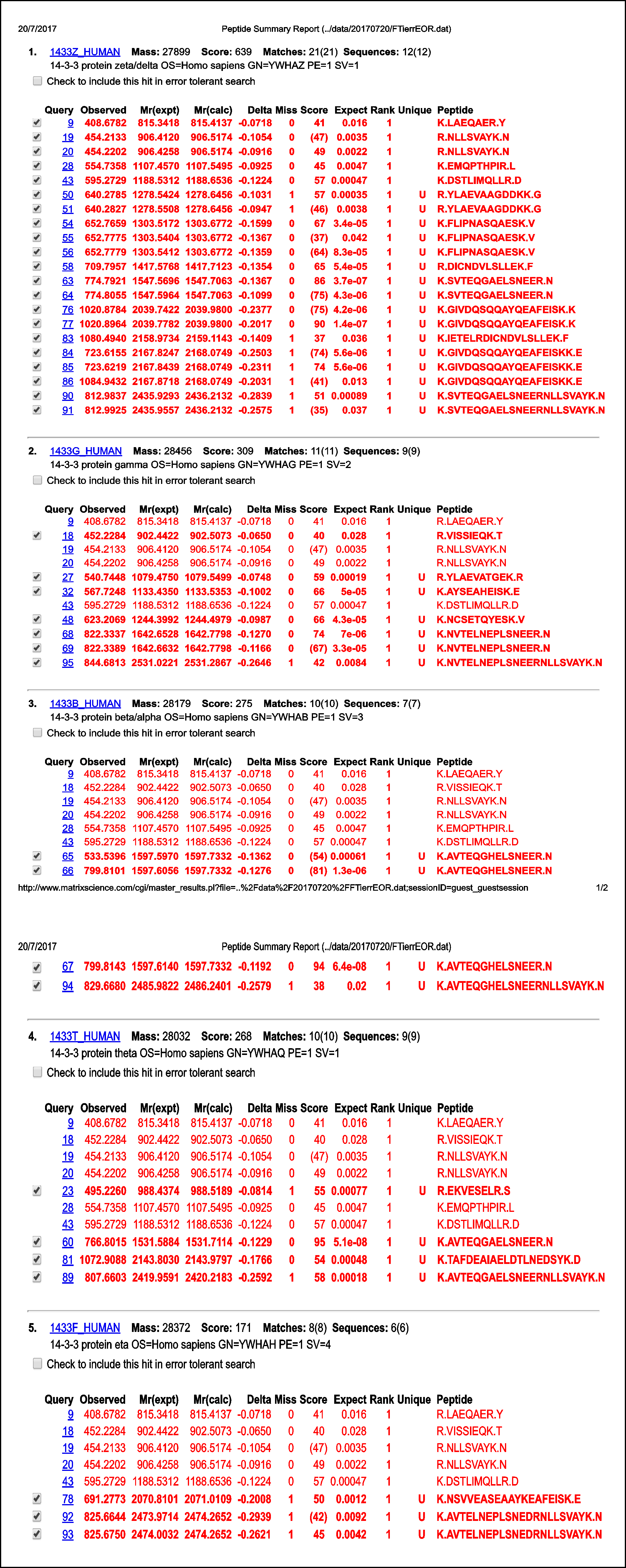

Supplement: S1 Fig — The protein assignment performed according to the highest number of covering peptides, in this case the 14-3-3 zeta/delta. However, the same spot also returned as 14-3-3 gamma, beta/alpha, theta and eta. The western blot unlatching results may be due to the different isoform of the tested antibody. (TIF) [file pone.0195388.s001.tif]

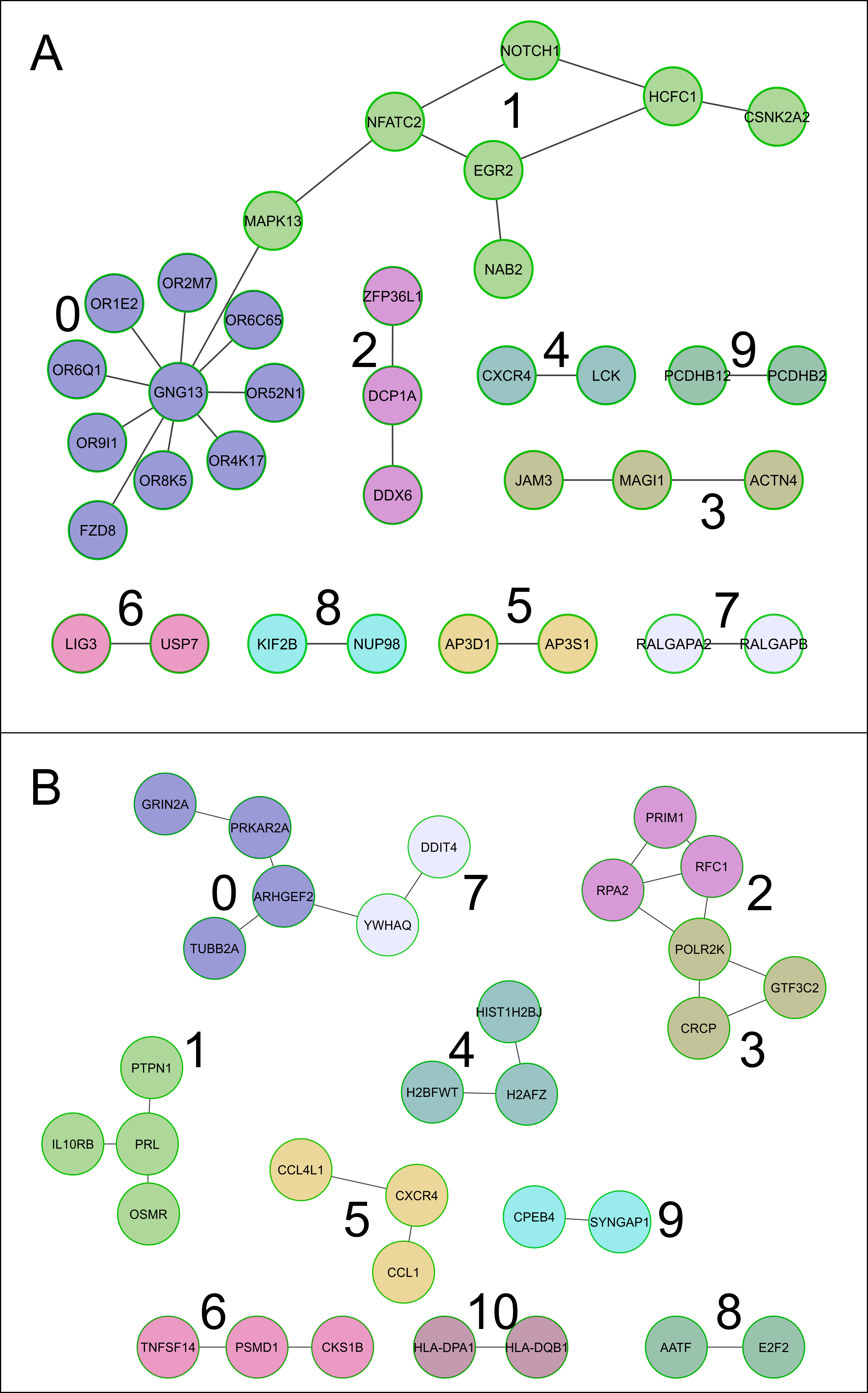

Supplement: S2 Fig — (A-B) Reactome FI networks derived by Dexa modulated genes in WT238 sample (A) and in A-T129RM (B). The gene expression analysis using the Affymetrix platform allowed us to isolate statistically and differentially expressed transcripts. The full list of differentially expressed transcripts is reported in S3 Supplementary File. The nodes colours represent the Reactome FI clustered genes while the numbers state the enrichment pathways of nodes in clusters as reported in S4 Supplementary File alongside biological pathways and molecular functions. (TIF) [file pone.0195388.s002.tif]

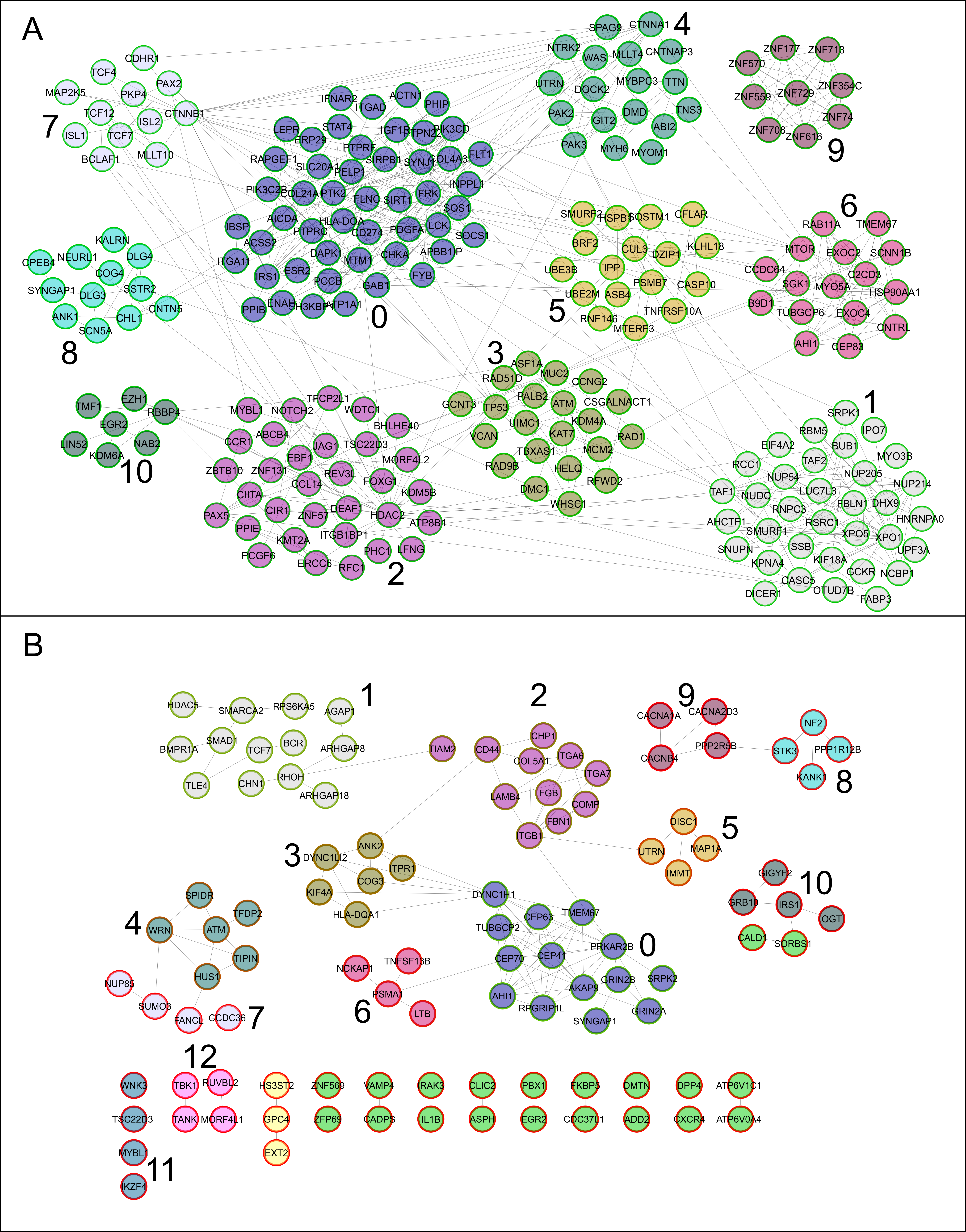

Supplement: S3 Fig — The STRING analysis of 2DE characterised spots from the A-T AT129RM sample lied to perform a splicing analysis of the same sample using the Affimetrix platform. Actually 614 transcripts proved to be alternatively spliced and were used to draw the functional network reported in B. Concurrently also the WT spliced transcripts were inferred and used to compute the functional network reported in A. The nodes colours represent the Reactome FI clustered genes while the numbers state the enrichment pathways of nodes in clusters as reported in S5 Supplementary File alongside biological pathways and molecular functions. (TIF) [file pone.0195388.s003.tif]

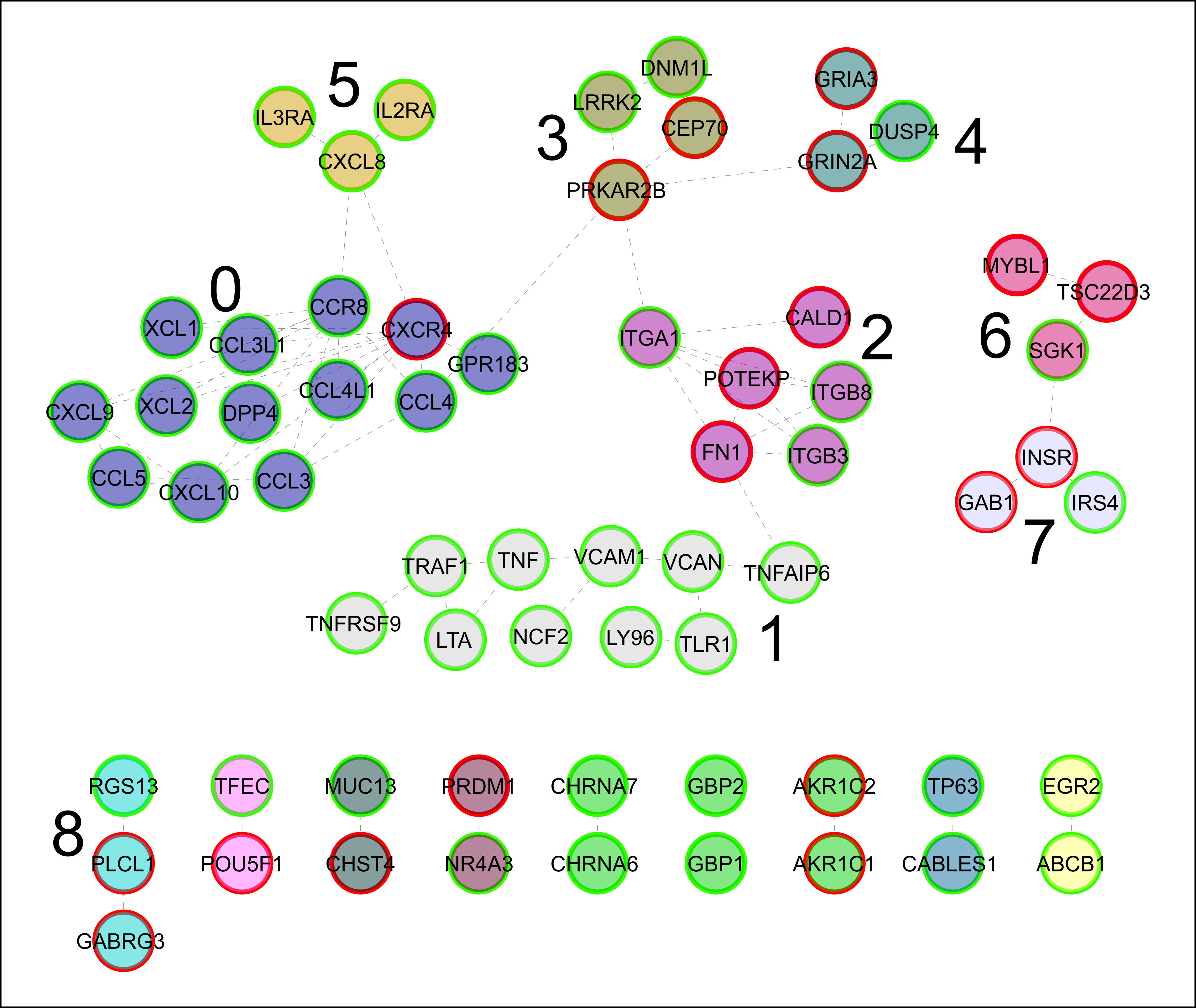

Supplement: S4 Fig — The network details are reported in S6 Supplementary File. The nodes colours represent the Reactome FI clustered genes while the numbers state the enrichment pathways of nodes in clusters. The profile of all A-T allowed the HLC outcome and sample clusterization illustrated in Fig 7. The whole A-T transcriptome statistically would decrease the Dexa modulated genes variance due to the genetic variability of the samples. (TIF) [file pone.0195388.s004.tif]
