# Supplementary material for: Proteomics and transcriptomics analyses of ataxia telangiectasia cells treated with Dexamethasone
Source: PLoS One. 2018 Apr 2;13(4):e0195388. doi: 10.1371/journal.pone.0195388 (PMC5880408; doi:10.1371/journal.pone.0195388)
Supplement: S1 Table — (DOCX) [file pone.0195388.s011.docx]

| Gene Symbol | |
| --- | --- |
| AT129RM | **WT238** |
| AP3S1 | AP3S1 |
| CHST4 | ARHGAP18 |
| CXCR4 | C12ORF77 |
| FAM107A | C8ORF87 |
| FKBP5 | CHI3L2 |
| FRMD3 | EGR2 |
| GRIN2A | FABP3 |
| GSAP | FAM25A |
| KDM2B | FCHSD2 |
| LCE3C | HSPB1 |
| MYO5C | IPO7 |
| NEK3 | LCK |
| PIK3IP1 | LOC101930011 |
| PNPLA8 | MEI1 |
| SLC44A1 | MTHFR |
| SYNGAP1 | MYO5A |
| TCF7 | NAB2 |
| VAMP5 | NAV2 |
| ZFY-AS1 | PLAGL1 |
|  | RALGAPA2 |
|  | SMC6 |
|  | TSC22D3 |
|  | VPS53 |
|  | WARS |
