# Supplementary material for: Proteomics and transcriptomics analyses of ataxia telangiectasia cells treated with Dexamethasone
Source: PLoS One. 2018 Apr 2;13(4):e0195388. doi: 10.1371/journal.pone.0195388 (PMC5880408; doi:10.1371/journal.pone.0195388)
Supplement: S2 Table — (DOCX) [file pone.0195388.s012.docx]

| **Common gene symbol between patients and LCLs microarray results.** | **Common pathways in Reactome FI networks obtained by between patients and LCLs microarray** |
| --- | --- |
| ABCA6 | Phosphatidylinositol 3-kinase binding |
| CXCR4 |  |
| FGL2 | Neurotransmitter binding |
| GBP1 |  |
| GBP2 | GPCR downstream signaling(R) |
| GBP5 |  |
| GPR137B | GPCR ligand binding(R) |
| GPR183 |  |
| HECW2 | Atypical NF-kappaB pathway(N) |
| IRS4 |  |
| ITGB3 | Inflammation mediated by chemokine and cytokine signaling pathway(P) |
| KLRB1 |  |
| KMO | Chemokine signaling pathway(K) |
| MS4A7 |  |
| OR4C3 | a6b1 and a6b4 Integrin signaling(N) |
| PEX5L |  |
| PRLR | Trk receptor signaling mediated by PI3K and PLC-gamma(N) |
| SAMD9L |  |
| SNX30 | IL12-mediated signaling events(N) |
| TMEM257 |  |
| TNF |  |
| TSC22D3 |  |
| UBASH3B |  |
| VCAM1 |  |
| VCAN |  |
| ZNF385D-AS1 |  |
